# Cerebrospinal Fluid Leaks and Pseudomeningocele after Posterior Fossa Surgery: Effect of an Autospray Dural Sealant

**DOI:** 10.7759/cureus.8379

**Published:** 2020-05-31

**Authors:** Young M Lee, Angel Ordaz, Beata Durcanova, Jennifer A Viner, Philip V Theodosopoulos, Manish K Aghi, Michael W. McDermott

**Affiliations:** 1 Neurological Surgery, University of California, San Francisco, USA

**Keywords:** pseudomeningocele, posterior fossa surgery, cost analysis, dural sealant

## Abstract

Background

Posterior fossa craniotomies can be complicated by cerebrospinal fluid (CSF) leaks, infection, meningitis, neurologic deficits, and intracranial hypotension caused by defective closure of the dura. Secondary dural closures such as pericranial graft, muscle graft, glue, sealants, or fat graft are used. However, there have been few studies examining the use of sealants with a polyethylene glycol and polyethylenimine component.

Objective

We studied the effect of one such sealant, Adherus® (HyperBranch Medical Technology, Durham, NC, USA), as an adjunct to secondary closure methods in the reduction of the use of abdominal fat grafting and lumbar puncture/drains.

Methods

We retrospectively reviewed the surgical records of all patients undergoing posterior fossa cranial surgery during a two-year period at a tertiary university affiliated medical center.

Results

Overall, data a total of 122 patients (62 in the no Adherus and 60 in the Adherus group) were collected. There was no statistically significant difference in the 30-day incisional CSF leak rate (4.1% vs. 6.5%; p=0.183), 30-day non-incisional CSF leak rate (11.3% vs. 5.0%; p=0.205), and 30-day pseudomeningocele rate (16.1% vs. 13.3%; p=0.663) in the no Adherus and Adherus groups, respectively. However, there was a significant reduction in the use of abdominal fat grafting (0% vs. 30.7%; p<0.001) and intraoperative CSF diversion techniques (58.1% vs. 23.3%; p<0.001). Every instance of the use of Adherus saved on average, $809.36.

Conclusions

A statistically significant reduction in the use of CSF shunting procedures during posterior fossa craniotomy/craniectomy was achieved after the introduction of Adherus with no increase in CSF leak rate.

## Introduction

Posterior fossa craniotomies can be complicated by cerebrospinal fluid (CSF) leak, infection, meningitis, neurologic deficits, and intracranial hypotension, which may be caused by defective closure of the dura. The standard method of dural closure involves the use of interrupted and/or running suture to appose the edges of the dura together to create a watertight seal. In addition, if a dural defect is noted with too much tension required for apposition of the dura, secondary dural closure with the use of pericranium, muscle, glue, sealants, or fat graft has been performed to augment the closure. In addition, biologic and synthetic compounds such as fibrin glue or polyethylene glycol (PEG) sealants can be used.

There is evidence to suggest that PEG sealants reduce CSF leak rates [1-3]. However, there have been a few studies examining the use of sealants with a PEG and polyethylenimine (PEI) component. One such dural sealant is Adherus® (HyperBranch Medical Technology, Durham, NC, USA), which is an FDA-approved product for use during brain surgery in patients aged 13 years or older intended to aid in preventing CSF leakage along the sutures by forming a watertight closure. It is composed of a PEG ester solution and a PEI solution, which is kept separate and when mixed together forms a sealant gel that is reabsorbed in approximately 90 days. Contraindications to its use include the use in confined spaces where nerves are present due to a risk of nerve compression from sealant gel swelling, which may increase by around 46% with application.

A previous study reported on the use of Adherus in 11 cases of intraoperative CSF leak treated with transphenoidal endoscopic surgery. There were no major complications, no postoperative CSF leaks, and no infections or other complications [4]. In addition, no inflammatory of healing problems were noted in routine follow-up. However, further studies on the use of Adherus and its effect on CSF leak rates are lacking in the literature.

The aim of this study was to report on the use of Adherus as an adjunct to primary closure methods, specifically to reduce the need for the use of abdominal fat grafting and lumbar puncture/drains and on CSF leak rates after posterior fossa cranial surgery.

## Materials and methods

Population

We retrospectively reviewed the surgical records of all patients undergoing posterior fossa cranial surgery during a two-year period at a tertiary academic medical center. Institutional Review Board (IRB) approval from the University of California San Francisco (UCSF) was obtained. Patient consent for the study was not obtained due to the purely observational nature of the study. The senior author proposed the use of the dural sealant for eliminating a secondary surgical procedure for the harvesting of abdominal fat graft. Posterior fossa cases were divided into two groups: a group in which Adherus dural sealant was used for dural closure and a control group in which Adherus was not used. During the second year of the two-year period, Adherus was used in all posterior fossa craniotomies and therefore the two groups were split based on whether the surgery was performed during the first or second year of the two-year period. There were three surgeons who operated on patients in both groups of this study. We retrospectively selected a total of 122 posterior fossa surgeries. Adherus dural sealant was used in 60 (49.2%) of these cases.

Additional demographic data were collected, including age, sex, smoking history, etiology of craniotomy, prior surgery, and the presence of diabetes. Other variables collected included the posterior fossa craniotomy approach and whether the patient required intraoperative CSF diversion, postoperative CSF diversion, and intraoperative abdominal fat grafting for dural closure. Outcome variables included 30-day postoperative incidence of incisional CSF leak, non-incisional CSF leak (otorrhea, rhinorrhea), and pseudomeningocele, as well as length of stay postoperatively.

Costs

Since the use of the dural sealant was proposed instead of harvesting of abdominal fat graft, an estimate of costs for the sealant was compared with that of the fat graft (Table 1). The cost of Adherus dural sealant and related supplies were obtained from the UCSF billing department and was determined to be $850. Costs related to abdominal fat grafting included 3-0 Vicryl® suture (Ethicon Inc., Somerville, NJ, USA; $11.82), 4-0 Monocryl® suture (Ethicon Inc.; $5.39), Mastisol® vial (Eloquest Healthcare, Ferndale, MI, USA; $1.66), Steri-StripsTM (3M, St. Paul, MN, USA; $0.78), and the allowable billable charge, which was listed at $2,270 in addition to 15 minutes of the operating room (OR) time and anesthesia time, which was estimated at $1,200 and $145, respectively. Thus, the total cost of Adherus was determined to be $850 versus the total cost of abdominal fat grafting of $3,634.65. The cost of abdominal fat graft wound site infection was also calculated. The professional charge for wound revision was $1,205 in addition to one hour of anesthesia time ($580) and one hour of OR time ($4,800). The average cost of hospital stay for wound infection was estimated to be $10,400 according to the publication Costs for Hospital Stays in the United States, 2012, by the Agency for Healthcare Research and Quality. This resulted in a total estimated cost of abdominal wound infection per patient of $16,985.

**Table 1:** Calculation of Adherus versus Fat Graft OR, operating room

	Fat Graft	Adherus
Cost of dural sealant per case	$0	$850
Supplies related to fat graft		
3-0 Vicryl suture	$11.82	$0
4-0 Monocryl suture	$5.39	$0
Mastisol	$1.66	$0
Steri-Strips	$0.78	$0
Allowable billable charge of fat graft	$2270	$0
15 minutes of OR time ($80/minute)	$1200	$0
15 minutes of anesthesia time	$145	$0
Total	$3634.65	$850

Outcomes

Incisional CSF leak was defined as fluid leakage from the surgical incision within 30 days after surgery that required clinical intervention. Non-incisional CSF leak was defined as otorrhea or rhinorrhea within 30 days after surgery that necessitated clinical intervention. Pseudomeningoceles were defined as clinically symptomatic fluid collections under the skin surrounding the surgical site, also within 30 days after surgery. Sub-clinical pseudomeningoceles identified by radiology were not included in the analysis. Re-admissions secondary to abdominal wound infections at abdominal graft site were also included.

Statistical analysis

Descriptive variables were presented using means and standard deviations (SDs). Demographic characteristics and outcome measures between the Adherus and control groups were compared using bivariate analysis. A chi-square test of independence was used to determine whether significant differences in means existed in categorical demographic and outcome variables between the Adherus and control groups. For continuous demographics or outcomes, an independent-samples t-test was used to compare means between the two groups. There were no significant differences found in any of the primary outcomes when compared between groups using bivariate analysis, and thus multivariate analysis was not performed.

## Results

Demographic data are summarized in Table 2. The mean age at surgery was 48.9 years (SD: 14.3), with no significant difference between the Adherus and no Adherus groups. Similarly, there was no significant difference in gender. The total patient population consisted of 49 men (40.2%) and 73 women (59.8%). The etiology of craniotomy had equal distribution as well, with a total of 93 (76.2%) tumors, 20 (16.4%) cysts, 5 (4.1%) Chiari malformations, 2 (1.6%) decompressions, and 2 (1.6%) inflammations. Furthermore, the frequency of the type of posterior fossa craniotomy performed was comparable. Retrosigmoid approach was used in 50 (41%) patients, whereas 63 (51.6%) patients underwent suboccipital craniotomy and 9 (7.4%) underwent other types of posterior fossa craniotomies. There was no significant difference between the Adherus and no Adherus groups in terms of a positive history of diabetes (7 patients; 5.7%), current smoking (10 patients; 8.2%), and former smoking (11 patients; 9%). There was significantly less use of postoperative CSF diversion methods, either LSAD (lumbar subarachnoid drain) or EVD (external ventricular drain) (31 patients in total, 25.4%) in the Adherus group when compared to the no Adherus group (33.9% vs. 16.7%; p=0.029). There was no difference in the number of patients with a history of same-site prior surgery (14 patients in total, 11.5%). The two groups did significantly differ in the use of intraoperative CSF diversion and intraoperative abdominal fat grafting. In the no Adherus group, 36 (58.1%) patients underwent CSF diversion during the procedure, whereas only 14 (23.3%) patients underwent CSF diversion during the procedure in the Adherus group (p<0.001). The diversion techniques included lumbar drain, lumbar puncture, or EVD. In the no Adherus group, 16 (25.8%), 15 (24.2%), and 5 (8.1%) patients underwent lumbar drain, lumbar puncture, and EVD, respectively, whereas, in the Adherus group, 2 (3.3%), 4 (6.7%), and 8 (13.3%) patients underwent lumbar drain, lumbar puncture, and EVD, respectively. Finally, abdominal fat grafting was performed in 19 (15.6%) patients in the no Adherus group, whereas, none in the Adherus group underwent abdominal fat grafting (p<0.001).

**Table 2:** Demographics SD, standard deviation; CSF, cerebrospinal fluid; LSAD, lumbar subarachnoid drain; EVD, external ventricular drain

	All (n=122)	No Adherus (n=62)	Adherus (n=60)	p-Value
Age at shunt placement, mean (SD)	48.9 (14.3)	48.1 (15.4)	49.7 (13.1)	0.536
Sex, n (%)				
Male	49 (40.2%)	27 (43.6%)	22 (36.7%)	
Female	73 (59.8%)	35 (56.5%)	38 (63.3%)	0.438
Etiology of craniotomy, n (%)				
Tumor	93 (76.2%)	45 (72.6%)	48 (80.0%)	
Cyst	20 (16.4%)	12 (19.4%)	8 (13.3%)	
Chiari malformations	5 (4.1%)	4 (6.5%)	1 (1.7%)	
Decompression	2 (1.6%)	1 (1.6%)	1 (1.7%)	
Inflammation	2 (1.6%)	0 (0.0%	2 (3.3%)	0.323
Type of posterior fossa craniotomy				
Retrosigmoid	50 (41.0%)	26 (41.9%)	24 (40.0%)	
Suboccipital	63 (51.6%)	29 (46.8%)	34 (56.7%)	
Other	9 (7.4%)	7 (11.3%)	2 (3.3%)	0.079
History of diabetes, n (%)	7 (5.7%)	4 (6.5%)	3 (5.0%)	0.730
Current smoking, n (%)	10 (8.2%)	3 (4.8%)	7 (11.7%)	0.169
Former smoking, n (%)	11 (9.0%)	6 (9.7%)	5 (8.3%)	0.796
Intraoperative CSF diversion, n (%)	50 (41.0%)	36 (58.1%)	14 (23.3%)	<0.001
Lumbar drain	18 (14.8%)	16 (25.8%)	2 (3.3%)	
Lumbar puncture	19 (15.6%)	15 (24.2%)	4 (6.7%)	
External ventricular drain	13 (10.7%)	5 (8.1%)	8 (13.3%)	
None	72 (59.0%	26 (41.9%)	46 (76.7%)	<0.001
Postoperative CSF diversion (LSAD/EVD), n (%)	31 (25.4%)	21 (33.9%)	10 (16.7%)	0.029
Intraoperative abdominal fat grafting procedure, n (%)	19 (15.6%)	19 (30.7%)	0 (0.0%)	<0.001
Prior surgery, n (%)	14 (11.5%)	8 (12.9%)	6 (10.0%)	0.615

Outcomes of the study are summarized in Table 3. The two groups did not differ in any of the assessments of outcomes performed in this study. In the no Adherus group, four (6.5%) patients experienced incisional CSF leak within 30 days, whereas, in the Adherus group, only one (1.7%) patient experienced incisional CSF leak within 30 days (p=0.183). Non-incisional CSF leak within 30 days occurred in seven (11.3%) patients in the no Adherus group and three (5.0%) the Adherus group (p= 0.205). Pseudomeningocele within 30 days was noted in 10 (16.1%) patients in the no Adherus group and 8 (13.3%) in the Adherus group p=0.663).

**Table 3:** Outcomes CSF, cerebrospinal fluid

	All (n=122)	No Adherus (n=62)	Adherus (n=60)	p-Value
Incisional CSF leak within 30 days, n (%)	5 (4.1%)	4 (6.5%)	1 (1.7%)	0.183
Non-incisional CSF leak (otorrhea, rhinorrhea) within 30 days, n (%)	10 (8.2%)	7 (11.3%)	3 (5.0%)	0.205
Pseudomeningocele within 30 days, n (%)	18 (14.8%)	10 (16.1%)	8 (13.3%)	0.663
Postoperative abdominal fat graft wound infection (requiring wound revision and readmission)	2 (1.6%)	2 (3.2%)	0 (0.0%)	0.161

Cost analysis is summarized in Table 4. Given the rates of abdominal fat grafting of 30.7% in the no Adherus group and 0% in the Adherus group, the total cost of abdominal fat grafting per 100 cases extrapolated was determined to be $56,700.54 overall, with $111,583.76 in the no Adherus group and $0 in the Adherus group. The cost of Adherus for 100 cases was $85,000. Thus, the total cost of procedures related to CSF leak prevention using abdominal fat grafting without Adherus was $111,583.76 per 100 cases and with Adherus was $85,000 per 100 cases. When the cost of abdominal fat graft infectious was included (two complications in the no Adherus group, with re-admission for washout), the total cost in the no Adherus group was $165,935.76, which resulted in a per-case savings of $809.36 (or $80,935.76 per 100 cases).

**Table 4:** Cost Savings of Adherus LOS, length of hospital stay

	All (n=122)	No Adherus (n=62)	Adherus (n=60)	p
% of Intraoperative abdominal fat grafting	15.6%	30.7%	0%	<0.001
Cost of abdominal fat grafting per 100 cases	$56700.54	$111583.76	$0	<0.001
% postoperative abdominal fat graft infection requiring revision and readmission (LOS > 2 days)	1.5	3.2	0	0.240
Cost of wound revision related to fat graft	$25477.50	$54352	$0	0.161
Cost of Adherus per 100 cases		$0	$85000	
Total cost of Adherus and fat grafting per 100 cases		$165935.76	$85000	
Cost savings per 100 cases		-$80935.76	$80935.76	
Cost saving per case		-$809.36	$809.36	

## Discussion

This case-control retrospective analysis explores the effects of the use of dural sealant on the operative techniques and subsequent outcomes of patients undergoing posterior fossa craniotomy. At our institution, Adherus was introduced with the intent of providing a cost-saving measure with a conscious decision to decrease the use of intra- and postoperative CSF diversion procedures and abdominal fat grafting. Until recently, the use of abdominal fat at the site of surgical defect has been considered the standard of care method for the reduction of postoperative CSF leaks after retrosigmoid, retrolabyrinthine, and translabyrinthine procedures in the posterior fossa. Despite its use, the CSF leak rate in the literature remains to be 6 to 17% [5-7]. With the introduction of Adherus, there was a conscious decision to decrease the use of abdominal fat grafting in retrosigmoid cases, and despite this, there was no significant increase in the rate of incisional CSF leak within 30 days (1.7% in the Adherus group and 6.5% in the no Adherus group) or formation of pseudomeningocele (13.3% in the Adherus group and 16.1% in the no Adherus group). Abdominal fat grafting, while considered a relatively minor procedure, is not without its complications. Two patients (data not shown) in the abdominal fat graft group experienced complications (one case of wound infection requiring washout and one case of delayed wound healing requiring revision), which is consistent with complications reported in the literature, including fat necrosis, wound infection, and abdominal hemorrhage [8]. Although there was insufficient power to detect a difference in abdominal fat graft related complications and no cost analysis was performed related to the abdominal fat graft procedure, the use of abdominal fat grafting significantly decreased after Adherus use (30.6% of patients) versus before Adherus use (6.3% of patients, p=0.001), with no concomitant increase in CSF leak or pseudomeningocele complication rates.

In addition to abdominal fat grafting, the use of intraoperative CSF diversion is an adjunct technique to posterior fossa craniotomy intended to provide relaxation of neural structures for better surgical corridors and also to reduce the pressure of CSF to aid in dural closure and reduction of CSF leaks. It is a commonly used procedure for the reduction of intraoperative fluid leak rates not only in posterior fossa surgery but also in surgery for pituitary adenomas [9]. As with abdominal fat grafting, a decision was made to reduce the use of these techniques, including lumbar puncture, LSAD, and EVD for the purposes of CSF leak reduction after the introduction of Adherus. This is apparent in the statistically significant reduction in the use of intraoperative CSF diversion techniques before (58.1% of patients) and after the introduction of Adherus (23.3% of patients; p<0.001). Although there was no specific analysis related to complications of CSF diversion procedures in this study, previous studies have reported major complication rates related to lumbar puncture and drainage of 3.0% (symptomatic subdural or subarachnoid hemorrhage, meningitis, and retained catheter) and minor complication rates of 5.2% (nerve root irritation, low-pressure headache requiring premature removal of drain, and local infection) [10]. The introduction of Adherus allowed a reduction in the use of these techniques and their associated time, cost, and complications, with no detected increase in CSF leak rates.

Specifically, we performed a cost-of-use analysis, finding that the use of Adherus resulted in cost savings of $809.36 per case in billable charges to the patient. This was driven by the reduced need for abdominal fat grafting and also the prevention of any wound complications and further admissions required for abdominal wound breakdown. However, in addition to the modest savings of approximately $809.36 of billable charges per case, we believe that a more significant benefit is the avoidance of the complications related to an additional incision in the abdominal area and the additional time under anesthesia and complications that accompany an additional incision, which are not trivial should they occur. Additionally, the use of adjunct CSF diversion methods, such as lumbar drain and EVD, add costs related to length of hospital stay were not included in the analysis. Typically, the protocol with respect to lumbar drains and EVD involves continuous volume drainage over the course of at least three days, which adds at least two days of hospital stay to patient admissions.

Our study has several limitations. First, it was a retrospective study using patient chart review for the abstraction of data and outcomes. Data gathering and patient recruitment were not performed with a prospective study design in mind; rather, a case series of consecutive patients undergoing posterior fossa craniotomies before and after the introduction of Adherus was collected and represents a convenience sample. However, we believe that we have reduced bias by selecting all patients who were treated with Adherus (n=60) after April 20, 2016, and simply including all consecutive patients within the same time duration before the introduction of Adherus (n=62). Second, patients were not assessed at definite time points for the outcomes of interest. Surveillance for the outcomes of interest was determined from a pragmatic clinical standpoint by abstracting from the patient’s chart daily progress notes, imaging results, and clinic notes for CSF leak, pseudomeningocele, and need for permanent CSF diversion. This may have resulted in an underreporting of complications, which may have been present and detected using standardized research assessments but may not have risen to clinical significance. Additionally, patients lost to follow-up and those who may have received treatment for complications at external hospitals may have been underreported. However, we do not believe that there should have been a systematic bias with respect to underreporting of complications.

Despite these limitations, we present the first study to assess the effect of Adherus on the rates of CSF leak and pseudomeningocele after posterior fossa surgery. While we found no difference in these outcomes, a large and statistically significant reduction in adjunctive procedures including lumbar puncture, LSAD, and abdominal fat grafting for CSF leak reduction was achieved. Our study was not powered to detect differences in complication rates resulting from adjunctive procedures, but larger prospective studies should be performed to quantify these differences. It seems safe to conclude, however, that the use of Adherus allows the reduction of abdominal fat graft, lumbar puncture, and LSAD procedures, with no change in CSF leak or pseudomeningocele rates after posterior fossa craniotomy/craniectomy.

## Conclusions

A retrospective analysis of posterior fossa craniotomy/craniectomies for various pathologies was performed to determine whether there were statistically significant differences in CSF leak rates and associated factors after the introduction of Adherus dural sealant along with its impact on complication rates and cost-effectiveness. A statistically significant reduction in the use of intraoperative abdominal fat graft, lumbar puncture, and LSAD procedures during posterior fossa craniotomy/craniectomy was achieved after the introduction of Adherus for all procedures, with no increase in the 30-day CSF leak or pseudomeningocele rates.

## References

[1] Osbun JW, Ellenbogen RG, Chesnut RM (2012). A multicenter, single-blind, prospective randomized trial to evaluate the safety of a polyethylene glycol hydrogel (Duraseal Dural Sealant System) as a dural sealant in cranial surgery. World Neurosurg.

[2] Pomeranz S, Constantini S, Umansky F (1991). The use of fibrin sealant in cerebrospinal fluid leakage. Minim Invasive Neurosurg.

[3] Than KD, Baird CJ, Olivi A (2008). Polyethylene glycol hydrogel dural sealant may reduce incisional cerebrospinal fluid leak after posterior fossa surgery. Neurosurgery.

[4] Zoia C, Bongetta D, Lombardi F, Custodi VM, Pugliese R, Gaetani P (2020). First impressions about Adherus, a new dural sealant. J Appl Biomater Funct Mater.

[5] Becker SS, Jackler RK, Pitts LH (2003). Cerebrospinal fluid leak after acoustic neuroma surgery: a comparison of the translabyrinthine, middle fossa, and retrosigmoid approaches. Otol Neurotol.

[6] Brennan JW, Rowed DW, Nedzelski JM, Chen JM (2001). Cerebrospinal fluid leak after acoustic neuroma surgery: influence of tumor size and surgical approach on incidence and response to treatment. J Neurosurg.

[7] Fishman AJ, Marrinan MS, Golfinos JG, Cohen NL, Roland JT, Jr Jr (2004). Prevention and management of cerebrospinal fluid leak following vestibular schwannoma surgery. Laryngoscope.

[8] Di Vitantonio H, De Paulis D, Del Maestro M (2016). Dural repair using autologous fat: Our experience and review of the literature. Surg Neurol Int.

[9] Mehta GU, Oldfield EH (2012). Prevention of intraoperative cerebrospinal fluid leaks by lumbar cerebrospinal fluid drainage during surgery for pituitary macroadenomas. J Neurosurg.

[10] Governale LS, Fein N, Logsdon J, Black PM (2008). Techniques and complications of external lumbar drainage for normal pressure hydrocephalus. Neurosurgery.

